# Isolation of a novel glycyrrhizin metabolite as a causal candidate compound for pseudoaldosteronism

**DOI:** 10.1038/s41598-018-33834-9

**Published:** 2018-10-22

**Authors:** Osamu Morinaga, Kan’ichiro Ishiuchi, Takeshi Ohkita, Chuanting Tian, Asuka Hirasawa, Miaki Mitamura, Yasuhito Maki, Tomoya Yasujima, Hiroaki Yuasa, Toshiaki Makino

**Affiliations:** 1 Department of Natural Medicines, Daiichi University of Pharmacy, 22-1 Tamagawamachi, Minami-ku, Fukuoka, Japan; 2 Department of Pharmacognosy, Graduate School of Pharmaceutical Sciences, Nagoya City University, 3-1 Tanabe-Dori, Mizuho-ku Nagoya, Japan; 3 Department of Biopharmaceutics, Graduate School of Pharmaceutical Sciences, Nagoya City University, 3-1 Tanabe-Dori, Mizuho-ku Nagoya, Japan

## Abstract

Pseudoaldosteronism is a common adverse effect associated with traditional Japanese Kampo medicines. The pathogenesis is mainly caused by 3-monoglucuronyl glycyrrhetinic acid (3MGA), one of the metabolites of glycyrrhizin (GL) contained in licorice. We developed an anti-3MGA monoclonal antibody (MAb) and an ELISA system to easily detect 3MGA in the plasma and urine of the patients. However, we found that some metabolites of GL cross-reacted with this MAb. Mrp2-deficient Eisai Hyperbilirubinemia rats (EHBRs) were administered glycyrrhetinic acid (GA), and we isolated 22*α*-hydroxy-18*β*-glycyrrhetyl-3-*O*-sulfate-30-glucuronide (**1**) from the pooled urine with the guidance of positive immunostaining of eastern blot as the new metabolite of GL. The IC_50_ of **1** for type 2 11β-hydroxysteroid dehydrogenase (11*β*-HSD2) was 2.0 µM. Similar plasma concentrations of **1** and GA were observed 12 h after oral administration of GA to EHBR. Compound **1** was eliminated via urine, whereas GA was not. In Sprague–Dawley (SD) rats orally treated with GA, compound **1** was absent from both the plasma and the urine. Compound **1** was actively transported into cells *via* OAT1 and OAT3, whereas GA was not. Compound **1**, when produced in Mrp2-deficiency, represents a potential causative agent of pseudoaldosteronism, and might be used as a biomarker to prevent the adverse effect.

## Introduction

Pseudoaldosteronism is a common adverse effect of traditional Japanese Kampo medicine. Early detection of pseudoaldosteronism is critical to prevent disease aggravation caused by Kampo medication. Its pathogenesis, however, has large interindividual variability, with an unpredictable onset time.

**Figure 1:**
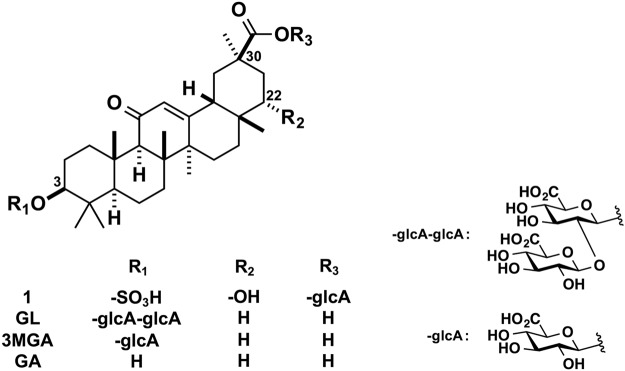
Chemical structures of **1**, GL, 3MGA, and GA.

Licorice, the roots of *Glycyrrhiza glabra* or *G. ularensis*, contains glycyrrhizin (GL), a glycoside of glycyrrhetinic acid (GA) with two molecules of glucuronic acid (Fig. 1). Licorice is prescribed frequently in Kampo medicine to treat a variety of diseases. GL is used to treat chronic hepatitis, gastric ulcers, and allergic diseases in Japan and Europe^1–3^, and as a natural sweetener in various foods. Licorice and the products containing GL can lead to pseudoaldosteronism, which is characterized by peripheral edema, hypokalemia, and hypertension^4^. GA, a major metabolite of GL, inhibits type 2 11*β*-hydroxysteroid dehydrogenase (11*β*-HSD2) in renal tubular epithelial cells, resulting in an elevation in cortisol levels, a potent agonist of mineralocorticoid receptors, leading ultimately to increased sodium retention and potassium excretion^5^.

A clinical study reported that the plasma concentrations of 3-monoglucuronyl-glycyrrhetinic acid (3MGA), another metabolite of GL (Fig. 1), was significantly higher in a patient group treated with GL with hypokalemia than in a group with normal potassium levels. The plasma GA concentration did not differ between the two groups, however^6^. In our previous reports, we showed that 3MGA was present in plasma and urine when multidrug resistant-association protein (Mrp) 2, a transporter involved in bile excretion, was impaired^7^. 3MGA is a substrate of the organic anion transporters (OATs) 1 and OAT3, and is actively transported from the plasma into tubular epithelial cells where it inhibits 11*β*-HSD2, although GA is not a substrate of these transporters^8^. Therefore, 3MGA may have a major role in pseudoaldosteronism caused by licorice and GL. Furthermore, plasma and urine 3MGA may be used as a biomarker to find early onset of pseudoaldosteronism.

3MGA was measured by using liquid chromatography-mass spectrometry (LC-MS) system. However, as licorice and GL products are not only used as foods but also as over-the-counter drugs, it is desirable to detect 3MGA in blood or urine very easily in pharmacy. In order to develop analysis kits for detecting 3MGA, we developed an anti-3MGA monoclonal antibody (MAb) and an enzyme-linked immunosorbent assay (ELISA) system. During this process, we found another novel metabolite of GL as a potent causal candidate of pseudoaldosteronism from urine of Mrp2-deficient Eisai Hyperbilirubinemia rats (EHBRs) treated with GA. We also reported the chemical structure of a novel GL metabolite, and elucidated its function in pseudoaldosteronism.

## Results

### Development of an anti-3MGA MAb and an ELISA system

**Figure 2:**
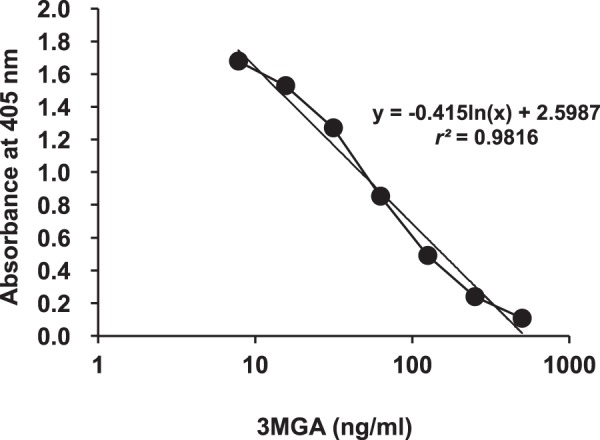
Profiles between the concentration of 3MGA and the absorbance at 405 nm in ELISA using anti-3MGA MAb and 3MGA-HSA conjugate as the immobilized antigen. Standard line was plotted by the absorbance at 405 nm against the logarithm of 3MGA concentration.

Here, we developed an anti-3MGA MAb and an ELISA system to measure 3MGA levels. Figure 2 shows the concentration profiles of 3MGA and absorbance in a competitive ELISA system by using anti-3MGA MAb. The detectable measurement range for 3MGA in this ELISA system was 0.010 µg/ml (0.015 µM)–0.50 µg/ml (0.75 µM). The isotype of anti-3MGA MAb was classified as IgG_2a_ with κ light chains. When the specificity of MAb to 3MGA was calibrated as 100%, the cross-reactivities to GA and GL were 1.04% and 0.22%, respectively, by ELISA. Regarding other terpenoid or flavonoid glycosides, the cross-reactivities of anti-3MGA MAb to licorice saponin G_2_, ginsenoside Rb_1_, saikosaponin a, paeoniflorin, and liquiritin were less than 0.02%.

**Figure 3:**
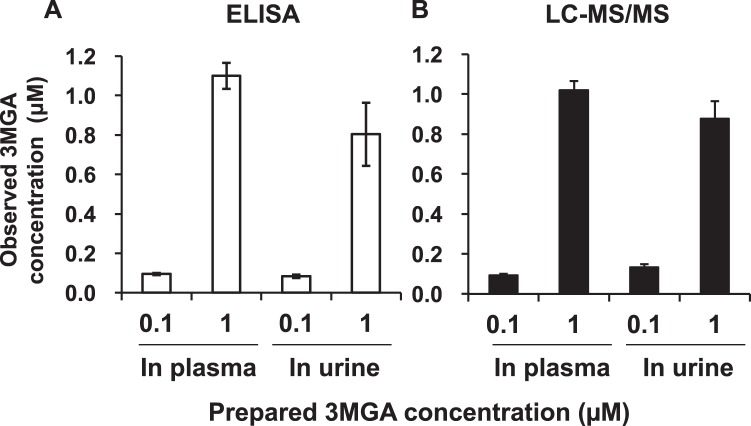
Comparison between ELISA using anti-3MGA MAb (left) and LC-MS/MS analysis (right) to measure the concentration of 3MGA in normal rat plasma and urine. Data are expressed as mean ± S.E. (*n* = 3).

Next, we prepared 3MGA solutions (0.10 and 1.0 µM) in normal rat plasma and urine, and measured the concentrations by using an ELISA system with anti-3MGA MAb and by LC-MS/MS analysis calibrated with 3MGA solutions in water. As shown in Fig. 3, both systems—ELISA and LC-MS/MS—could measure the concentrations of 3MGA in plasma and urine accurately.

### Pharmacokinetic profiles of 3MGA in EHBRs measured using anti-3MGA MAb

**Figure 4:**
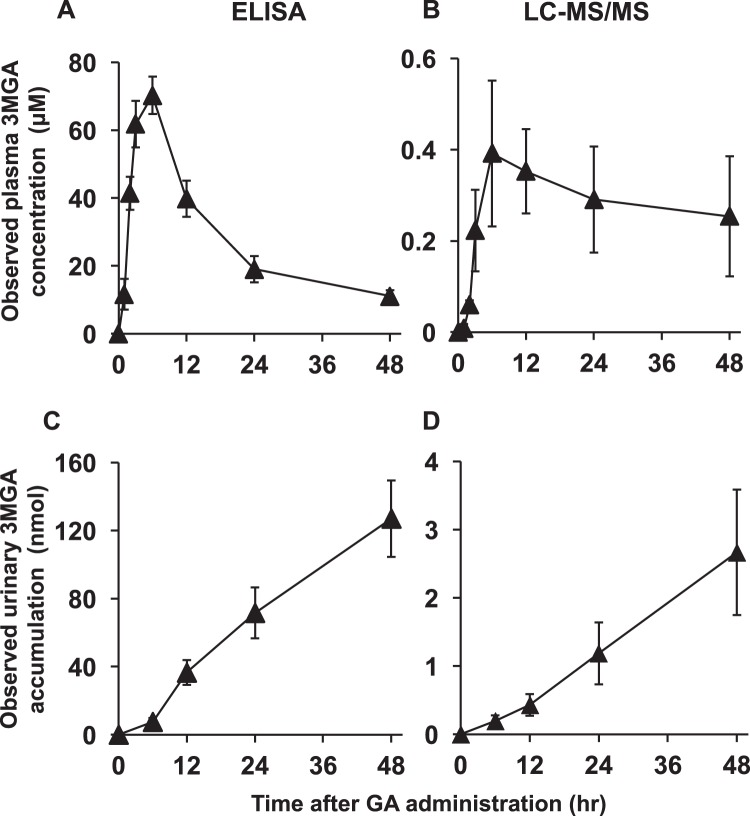
The profiles of observed plasma concentration (upper) and urinary elimination (lower) of 3MGA measured by ELISA (left) and LC-MS/MS (right) after oral administration of GA to female EHBRs. GA (50 mg/kg) was orally administered to female EHBRs, and the plasma and urine were collected. The concentrations of 3MGA were measured by ELISA using anti-3MGA MAb and LC-MS/MS, and observed data were plotted. Data are expressed as mean ± S.E. (*n* = 5–6).

We collected plasma and urine samples from female EHBRs orally administered with GA (50 mg/kg), and measured the concentrations of 3MGA by using ELISA and LC-MS/MS. Figure 4 shows the observed concentration profiles of 3MGA in plasma and urine samples of EHBRs after the treatment with GA. The plasma concentrations of 3MGA in EHBRs increased after oral treatment with GA, and the maximum concentration was observed at 6 h. Next, the concentrations of 3MGA gradually decreased, and 3MGA elimination via urine was detected, which increased up to 48 h. When comparing ELISA and LC-MS/MS, the observed values of 3MGA concentrations by ELISA were 40–100-fold higher than those measured by using LC-MS/MS, although the profiles were similar to one another.

### Isolation and structure elucidation of compound 1 from EHBR urine guided by eastern blot analysis

Female EHBRs were administered 1 mg/ml GA via drinking water for 3 months, and their urine was collected and pooled. Next, we conducted eastern blot analysis using anti-3MGA MAb for the detection of 3MGA and other compounds which have related structure in urine sample. Urine or 3MGA standard solution was spotted onto a thin layer chromatography (TLC) plate, developed using the solvent, transferred, and fixed onto a polyethersulfone (PES) membrane. The membrane was treated with anti-3MGA MAb, enzyme-labeled secondary antibody, and its substrate 4-chloro-1-naphthol. The positive staining spots were appeared other than 3MGA, which position was more hydrophilic than 3MGA.

**Figure 5:**
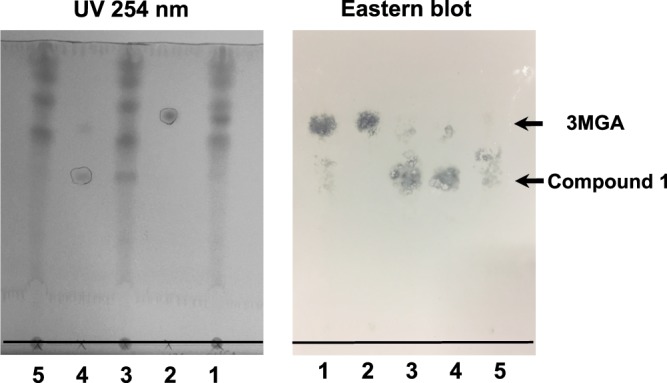
Eastern blot analysis of urine collected from EHBRs orally treated with GA using anti-3MGA MAb. On the photographs, base lines were added. On TLC plate, the following samples were spotted at the base line; lane **1**, double spot of 6 µl of urine collected from EHBRs which were orally administered with GA as drinking water (1 mg/ml) and 2 µl of 3MGA (1 mM); lane **2**, 2 µl of 3MGA (1 mM); lane **3**, double spot of 6 µl of urine collected from EHBRs and 2 µl of compound **1** (1 mM); lane **4**, 2 µl of compound **1** (1 mM); lane **5**, 6 µl of urine collected from EHBRs. Then, the spots were spreaded out using H_2_O/BuOH/AcOH (2:7:3), and the top line of the solvent was penciled. Photograph of TLC plate detected by UV absorption at 254 nm (left) was taken. On the lanes **2** and **4** used as positive control, the spots appeared were marked by pencil. The spots on the plate were transferred onto PES membrane, fixed, blocked, and strained by anti-3MGA MAb. Photograph of the membrane was taken (right). *Rf* values of 3MGA and compound **1** were 0.76 and 0.50 respectively.

From 1 liter of urine collected from EHBRs orally treated with GA, 3.5 mg of compound **1** was successfully isolated using the guidance of eastern blot. Figure 5 shows TLC pattern using UV absorption and eastern blot profile for the standard solution of 3MGA, urine sample, and isolated compound **1**.

Compound **1** [[α]_D_^23^ +33 (*c* 1.0, MeOH)] exhibited a deprotonated molecule at *m/z* 741 (M-H)^−^ in the ESIMS, and the molecular formula, C_36_H_54_O_14_S, was established by HRESIMS [*m/z* 741.3153, (M-H)^−^, *Δ*-0.3 mmu]. ^1^H and ^13^C NMR data (Table 1) and the HSQC spectrum of **1** revealed 36 carbon signals due to three carbonyl carbons, one sp^2^ nonprotonated carbon, one sp^2^ methines, six sp^3^ nonprotonated carbons, ten sp^3^ methines, eight sp^3^ methylenes, and seven sp^3^ methyl groups. Of these, two carbonyl carbons (*δ*_C_ 176.6 and 171.8) and six sp^3^ methines (*δ*_C_ 95.6, 87.4, 77.6, 77.4, 76.1, 73.6, and 72.9) were attributed to those attached to an oxygen atom. The ^1^H NMR spectrum of **1** was similar to that of GA, which implied that **1** was a metabolite of GA. A fragment peak at *m/z* 565 (M-H-C_6_H_8_O_6_)^−^ in the ESIMS of **1** indicated the presence of a glucuronic acid moiety. Furthermore, comparison between the molecular formulae of **1** and GA suggested that **1** possessed one hydroxy group and one sulfo group.

**Table 1:** ^1^H and ^13^C NMR Data (CD_3_OD) of compound **1**.

Position	*δ*_H_^a^	*δ*_C_^b^	HMBC
1a	2.74 (1H, d 13.5 Hz)	40.1	25
1b	1.04 (1H, nd^c^)		
2a	2.08 (1H, brd 11.5 Hz)	25.2	
2b	1.78 (1H, nd^c^)		
3	3.96 (1H, m)	87.4	23, 24
4		39.9	23, 24
5	0.86 (1H, nd^c^)	56.6	7, 23, 24, 25
6a	1.65 (1H, brd 13.5 Hz)	18.6	
6b	1.49 (1H, nd^c^)		
7a	1.76 (1H, nd^c^)	33.7	26
7b	1.45 (1H, nd^c^)		
8		46.7	9, 26, 27
9	2.47 (1H, s)	63.1	12, 25, 26
10		38.1	9, 25
11		202.5	9, 12
12	5.61 (1H, s)	129.2	18
13		171.1	12, 15b, 18, 19b, 27
14		45.1	12, 16a, 16b, 26, 27
15a	1.80 (1H, nd^c^)	27.2	27
15b	1.29 (1H, m)		
16a	1.80 (1H, nd^c^)	20.3	28
16b	1.48 (1H, nd^c^)		
17		38.4	15b, 16b, 19a, 21a, 28
18	2.21 (1H, brd 13.5 Hz)	49.0	12, 16a, 16b, 28
19a	1.89 (1H, brd 12.5 Hz)	41.6	29
19b	1.80 (1H, nd^c^)		
20		45.1	21a, 29
21a	2.16 (1H, brd 11.0 Hz)	39.5	
21b	1.51 (1H, nd^c^)		
22	3.42 (1H, nd^c^)	76.1	21a, 21b, 28
23	1.07 (3H, s)	28.7	24
24	0.86 (3H, s)	16.9	23
25	1.16 (3H, s)	17.0	5, 9
26	1.14 (3H, s)	19.2	9
27	1.44 (3H, s)	23.9	15b
28	0.93 (3H, s)	25.5	
29	1.25 (3H, s)	28.1	
30		176.6	19b, 21b, 29, 1′
1′	5.53 (1H, d 7.5 Hz)	95.6	2′
2′	3.42 (1H, nd^c^)	73.6	3′
3′	3.45 (1H, nd^c^)	77.6	2′
4′	3.60 (1H, dd 9.0, 9.0 Hz)	72.9	
5′	3.90 (1H, d 9.0 Hz)	77.4	1′, 4′
6′		171.8	4′

^a^500 MHz. ^b^125 MHz. ^c^nd: *J*-values were not determined because of overlapping with other signals.

**Figure 6:**
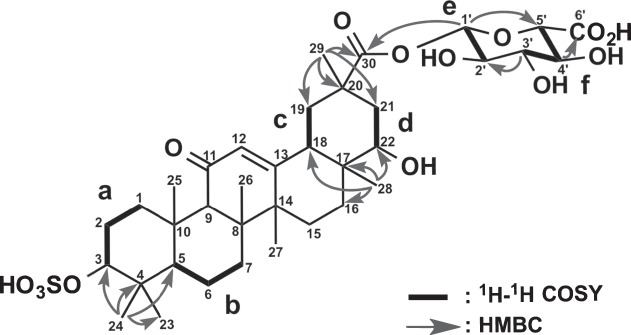
Selected 2D NMR correlations for compound **1**.

The planar structure of **1** was elucidated by analysis of 2D NMR data including the ^1^H-^1^H COSY, HSQC, and HMBC spectra in CD_3_OD. Six structural units **a** (C-1-C-3), **b** (C-5-C-7), **c** (C-18-C-19), **d** (C-21-C-22), **e** (C-1′-C-2′) and **f** (C-3′-C-5′) were disclosed by the ^1^H-^1^H COSY spectrum of **1** (Fig. 6). The analyses of HMBC spectra, especially a key HMBC correlation for H_3_-28 (*δ*_H_ 0.93) to C-22 (*δ*_C_ 76.1) (Fig. 6 and Table 1), revealed that the core skeleton of **1** was the same as that of an oxygenated GA at C-22. HMBC correlations for H-1′ (*δ*_H_ 5.53) to C-5′ (*δ*_C_ 77.4), H-3′ (*δ*_H_ 3.45) to C-2′ (*δ*_C_ 73.6), and H-4′ (*δ*_H_ 3.60) to C-6′ (*δ*_C_ 171.8) along with three proton coupling constants, ^3^*J*_H-1_′_/H-2_′(7.5 Hz), ^3^*J*_H-3_′_/H-4_′(8.8 Hz), and ^3^*J*_H-4_′_/H-5_′ values (8.8 Hz) indicated that units **e** and **f** formed a *β*-glucuronic acid group. An HMBC cross-peak of H-1′ to C-30 (*δ*_C_ 176.6) revealed that the glucuronic acid moiety was connected to GA between C-30 and C-1′ via an oxygen atom. Finally, comparison between the chemical shifts of C-3 (*δ*_H_ 3.96 and *δ*_C_ 87.4) and C-22 (*δ*_H_ 3.42 and *δ*_C_ 76.1) concluded that a sulfo group was connected to an oxygen atom at C-3. Thus, the planar structure of **1** was elucidated as shown in Fig. 6.

**Figure 7:**
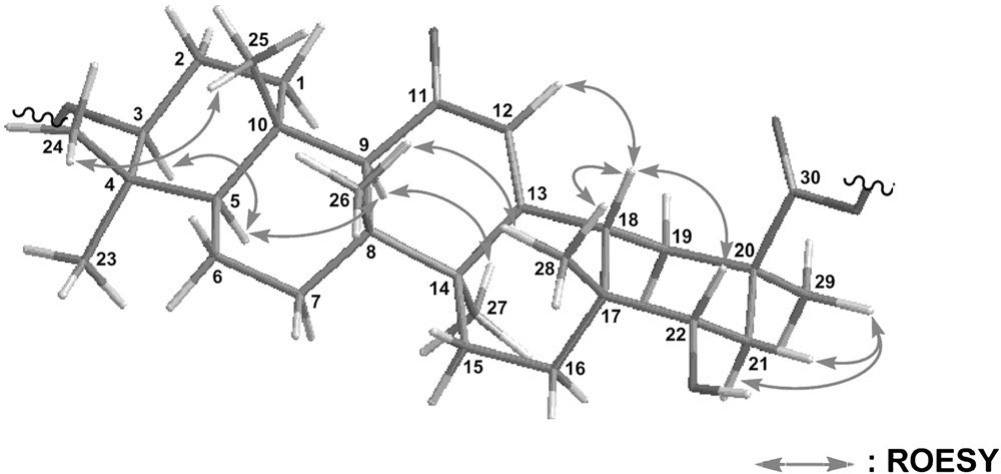
Selected NOESY correlations for compound **1**.

The relative stereochemistry of **1** was deduced from ROESY data (Fig. 7). ROESY correlations for H-3/H-5, H_3_-24/H_3_-25, H-9/H-5, and H_3_-27, H_3_-26/H_3_-28, H-18/H-12, H-22, and H_3_-28; and H_3_-29/H-21a and H-21b revealed that the stereochemistry of **1** was the same as that of 18*β*-GA except for C-22. The *α*-orientation of the hydroxy group at C-22 was revealed by a ROESY correlation for H-18/H-22. The relative stereochemistry of **1** was elucidated as shown Fig. 7. The CD spectrum of **1** showed a positive Cotton effect at 229 nm similar to that of GA. Thus, the absolute configuration of **1** was established as 3*S*, 5*R*, 8*R*, 9*R*, 10*S*, 14*S*, 17*R*, 18*S*, 20*R*, and 22*S*.

### Inhibitory effect of compound 1 on rat 11*β*-HSD2

**Figure 8:**
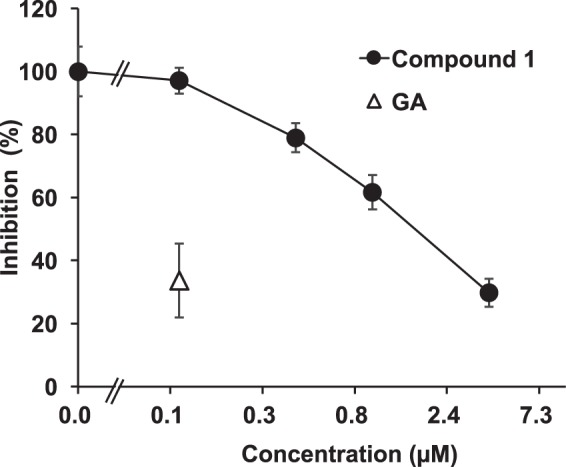
Inhibitory effects of compound **1** on 11*β*-HSD2 using rat kidney microsomes. [^3^H]cortisone and GA or compound **1** were mixed with rat kidney microsomes, and incubated at 37 °C for 30 min. Then, the amount of [^3^H]cortisol was measured. Data are expressed as mean ± S.E. (*n* = 4) of the percentage of the amount of [^3^H]cortisol in the mixture without samples.

We measured the inhibitory effect of compound **1** on 11*β*-HSD2 using kidney microsome fractions collected from male Wistar rats. As shown in Fig. 8, compound **1** inhibited 11*β*-HSD2 activity in a concentration-dependent manner, and its half-maximum inhibitory concentration (IC_50_) value was 2.0 µM.

### Pharmacokinetic profiles of compound 1 in EHBR treated with GA

**Figure 9:**
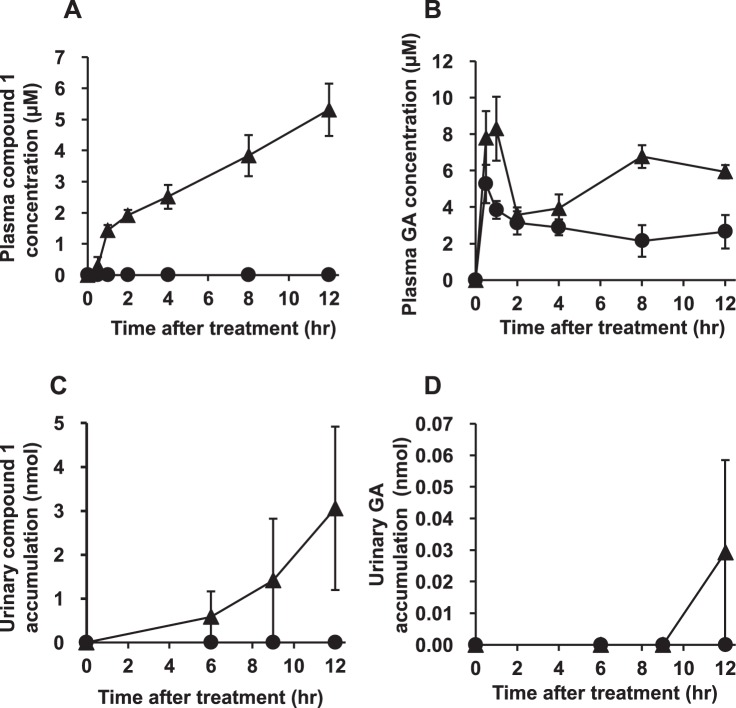
The plasma concentration (**A**,**B**) and urinary elimination (**C**,**D**) profiles of compound **1** (**A**,**C**) and GA (**B**,**D**) and in female SD rats (closed circle) and EHBRs (closed triangle) after oral administration of GA. GA (200 mg/kg) was administered orally to female SD rats or EHBRs, and plasma and urine samples were collected. The concentrations of compound **1** and GA were measured by LC-MS/MS. Data are plotted as mean ± S.E. (*n* = 4).

We collected plasma and urine samples from both female Sprague Dawley (SD) rats and EHBRs orally administered with GA (200 mg/kg), and measured the concentration of compound **1** by LC-MS/MS. Figure 9 shows the plasma concentration profiles and urinary eliminations data of compound **1** and GA in SD rats and EHBRs. In both SD rats and EHBRs, GA was appeared in plasma 30 min after the treatment, and the concentrations were sustained for 12 h. The plasma concentrations of GA in EHBRs were higher than those in SD rats. Urinary GA eliminations was almost undetectable levels in both SD rats and EHBRs. Regarding compound **1**, the plasma concentrations in EHBRs treated with GA remained stable for 12 h and was then eliminated via urine; however in SD rats, compound **1** was not detected in both plasma and urine. In plasma collected after 12 h of GA treatment from EHBRs, the concentration of compound **1** was 5.3 µM, and this value was similar to that of GA (5.9 µM).

### Uptakes of compound 1 by rat kidney slices and cells expressing OAT1 and 3

**Figure 10:**
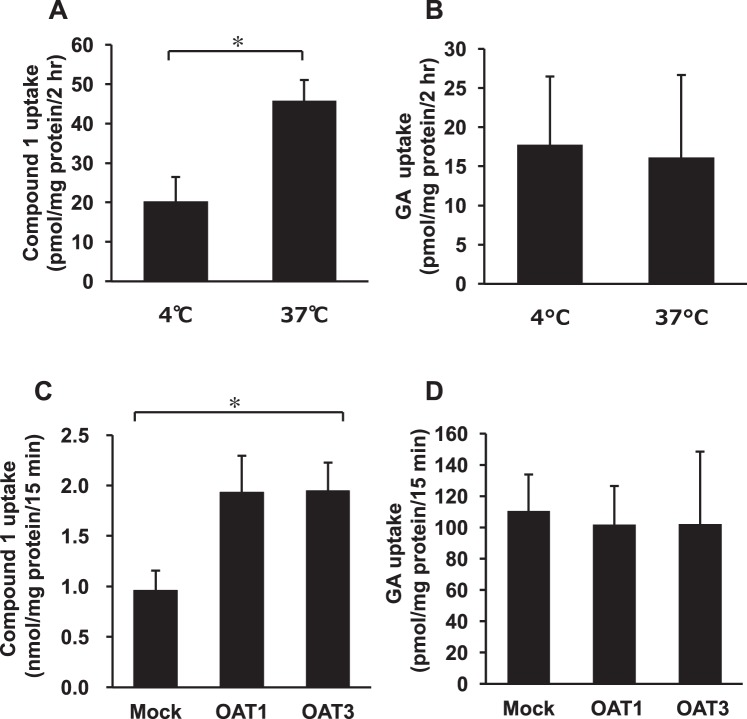
The uptake of compound **1** and GA into rat kidney slice (**A**,**B**) and MDCK II cells expressing OAT1 or 3 (**C**,**D**). GA (200 mg/kg) was administered orally to both sexes of EHBRs, and plasma was collected 12 hr after oral administration. The concentrations of compound **1** and GA in pooled plasma were 15 and 7.0 µM (female), and 79 and 3.2 µM (male), respectively. Rat kidney slices prepared from normal SD rats were incubated with pooled plasma of female EHBRs for 2 hr, and the tissue samples were homogenized. MDCK II cells stably expressing OAT1 or 3 were incubated with pooled plasma of female (for GA) or male (for compound **1**) EHBRs for 15 min, and the contents in the cells were extracted using ethanol. The concentrations of compound **1** and GA in the homogenates and the extracts were measured by LC-MS/MS. Data are plotted as mean ± S.E. (*n* = 3 for kidney slices of GA; *n* = 6 for kidney slices of compound **1**; *n* = 6 for cell experiments). ^*^P < 0.05 by Student’s *t*-test (**A**,**B**) or by one-way ANOVA (**C**,**D**).

As the amount of isolated compound **1** was small, the following experiments were conducted using pooled plasma samples collected from each female and male EHBR 12 h after oral treatment with GA (200 mg/kg). The concentrations of compound **1** and GA in pooled plasma collected from female EHBRs were 15 and 7.0 µM, respectively. Kidney slices were prepared from male SD rats, and incubated with pooled plasma collected from female EHBRs which pH was adjusted to 5.5 at 4 or 37 °C for 2 h. After washing, the kidney slices were homogenized, and the concentrations of compound **1** and GA in the homogenate were measured by using LC-MS/MS. Figure 10 A,B show the amount of compound **1** and GA in the homogenate of kidney slices. The uptakes of compound **1** by kidney slices incubated at 37 °C was significantly higher (*p* < 0.05) than the uptake by those incubated at 4 °C. On the contrary, the amount of GA in kidney slices was similar between samples incubated at 4 and 37 °C.

MDCK II cells stably expressing OAT1 and OAT3 were incubated with pooled plasma collected from female EHBRs which pH was adjusted to 5.5 at 37 °C for 15 min, and the uptake of compound **1** and GA by the cells was measured by using LC-MS/MS. Figure 10D shows the uptake of GA was similar in mock cells and the cells expressing OAT1 or 3, however, the uptake of compound **1** by cells were too low not to measure accurately. Therefore, we used pooled plasma sample collected from male EHBRs which contained 79 µM and 3.2 µM of compound **1** and GA, respectively. The uptakes of compound **1** by the cells expressing OAT1 and 3 were higher than that by mock cells, and ANOVA analysis exhibited statistically significance (*p* < 0.05) among 3 groups (Fig. 10C).

### Binding assay of compound 1 with serum albumin

The pooled plasma samples were ultracentrifuged at 1 × 10^4^ molecular weight cut off. The concentration of compound **1** in the filtrate was undetectable by LC-MS/MS analysis. The albumin-binding ratio of compound **1** in plasma was calculated to be more than 99.9%.

## Discussion

According to the previous studies^6–8^, 3MGA can be used as a biomarker to find the body constitution to cause pseudoaldosteronism in patients consuming licorice or a GL prescription. In this study, we developed an anti-3MGA MAb that can be used for ELISA. As the main metabolite of GL is GA^9^, anti-3MGA MAb can recognize GA and 3MGA separately. In order to produce a MAb that recognizes 3MGA selectively, we synthesized a compound with a hapten-carrier protein conjugate at the carboxylic acid, C-30, of 3MGA by carbodiimide method to bind KLH or HSA without the steric hindrance between 3MGA and carrier protein. Cross-reactivity of the anti-3MGA MAb to GA and GL was 1.04% and 0.22%, when the specificity to 3MGA was calibrated as 100%. From this result, the MAb could distinguish the sugar moieties at C-3 of 3MGA, although the aglycone part of GL and 3MGA was GA. Furthermore, we checked the cross-reactivities of the anti-3MGA MAb with natural products that have related structures, and found that this anti-3MGA MAb can be used for measuring 3MGA concentration.

Next, we developed a competitive ELISA system to measure 3MGA. When we prepared solutions of 3MGA with various concentration in H_2_O, and obtained a calibration line between the concentrations of 3MGA and the absorbances in this ELISA system, a strong correlation was observed. We prepared solutions of 3MGA with normal plasma and urine collected from SD rats and analyzed the concentrations using ELISA. The observed values were proportional to the observed LC-MS/MS values, suggesting that the anti-3MGA MAb did not cross-react with endogenous substances in normal rat plasma and urine.

However, when we measured the concentrations of 3MGA in plasma and urine collected from EHBRs orally administered GA by ELISA and LC-MS/MS, the observed values measured by ELISA were approximately 40–100-fold higher than those measured by LC-MS/MS. It is suggested that the anti-3MGA MAb might cross-react with other metabolites of GA present in plasma and urine of EHBRs orally administered with GA.

In order to identify the compounds with which anti-3MGA MAb cross-reacted, we employed an eastern blotting technique developed by Shoyama *et al*., to facilitate the immunostaining of low molecular-weight compounds on a polyvinyliden difluoride (PVDF) or PES membrane by using a special blotting system^10^. As small molecular compounds easily washed out of the membrane, fixation is necessary. We employed an 1-ethyl-3-(3′-dimethylaminopropyl) carbodiimide hydrochloride (EDC)/*N*-hydroxysuccinimide (NHS) method to link the carboxyl groups of small molecular compounds to the amine groups of proteins, as various types of hapten-carrier protein conjugates have been successfully prepared for eastern blotting^11,12^. It is predicted that the metabolites of GA would have a carboxyl group at C-30 or be the conjugates of glucuronic acid in the case of positive immunostaining spots by eastern blot analysis using anti-3MGA MAb. As expected, the positive staining spot that was distinct from that of standard 3MGA was found in the urine of EHBRs treated with GA, and the position of positive staining spot was more hydrophilic than 3MGA, whereas 3MGA was not detected in the urine sample. It is likely that the concentration of this metabolite in the urine of GA-treated EHBRs might be higher than that of 3MGA.

Next, we isolated the metabolites the cross-reacted with anti-3MGA MAb from the urine of EHBRs treated with GA by the guidance of eastern blotting positive staining, and the structure of compound **1** was elucidated to be 22*α*-hydroxy-18*β*-glycyrrhetyl-3-*O*-sulfate-30-glucuronide on the basis of spectroscopic data; **1** is a new metabolite of GA. 3MGA, 18*β*-glycyrrhetyl-30-glucuronide, and 18*β*-glycyrrhetyl-3-*O*-sulfate were previously reported as the metabolites of GA in humans^13^. This suggests that humans have sulfotransferase that conjugates sulfate with a hydroxy group at C-3 and glucuronyltransferase that conjugates glucuronic acid with a carboxylic acid group at C-30 of GA. Moreover, 22*α*-hydroxy-18*β*-glycyrrhetinic acid was found in the reaction mixture of rat liver microsomes and GA^14,15^. Hydroxylation of GA at C-22 could be catalyzed by some class of cytochrome P450 (CYP) expressed in liver. GA is a competitive inhibitor of CYP3A4, 2C9, and 2C19, with IC_50_ values of 8.2–14, 33–43, and 41–74 µM, respectively^16,17^. This suggests that hydroxylation of GA at C-22 may be catalyzed by CYP3A in rats.

Similar to the pharmacokinetic profile of 3MGA shown in our previous study^8^, compound **1** did not appear in the plasma of normal SD rats orally treated with GA, but appeared in that of EHBRs, although the plasma concentrations of GA were similar in SD rats and in EHBRs. This result suggests that compound **1** could be the substrate of Mrp2 and may be eliminated into bile in normal conditions.

Most of the GA metabolites in plasma were bound to albumin^18,19^, and the binding rate of compound **1** to albumin was more than 99.9%. Because albumin-bound compounds cannot penetrate through cellular membranes by passive diffusion, the compounds that can inhibit 11*β*-HSD2 *in vivo* must be recognized as the substrates of transporters that transport the compounds from blood into tubular cells. Compound **1** was demonstrated to be an OAT substrate using uptake assays in rat kidney slices and cells stably expressing OAT1 and 3. GA was not found to be an OAT substrate. These results can explain why GA was not detected in the urine; however, 3MGA and compound **1** were detected in the urine of EHBRs orally treated with GA. Our previous study revealed that the IC_50_ values of rat 11*β*-HSD2 activity were 2.2 µM for GL, 0.26 µM for 3MGA, and 0.32 µM for GA^8^, and the IC_50_ value of compound **1** was 2.0 µM. Therefore, the potency of compound **1** was similar to that of GL, and about 10-times lower than that of 3MGA and GA. However, as the plasma concentration of compound **1** was approximately 10-times higher than that of 3MGA in EHBRs orally treated with GA, compound **1** may also play important roles in 11*β*-HSD2 inhibition *in vivo*. The discovery of compound **1** indicated a possibility that there might be other GA metabolites as the causal candidates for licorice-induced pseudoaldosteronism, and the investigation of other GA metabolites is currently in progress.

In conclusion, by developing anti-3MGA MAb to detect 3MGA in plasma or urine, we discovered a new metabolite of GA, compound **1**, in Mrp2-deficient EHBRs, and elucidated the structure of **1** as 22*α*-hydroxy-18*β*-glycyrrhetyl-3-*O*-sulfate-30-glucuronide. Similar to 3MGA, compound **1** was not present in the plasma and urine of normal rats. Although the inhibitory effect of compound **1** on 11*β*-HSD2 was weaker than 3MGA, the plasma concentration of compound **1** was higher than that of 3MGA in EHBRs orally treated with GA. Not only 3MGA but also compound **1** could play important roles in 11*β*-HSD2 inhibition *in vivo*, and might be used as biomarkers to prevent pseudoaldsteronism when patients use licorice. Now we are analyzing the plasma and urinary concentrations of compound **1**, 3MGA and GA in the patients who have licorice-induced pseudoaldosteronism, and the results will be described in our future articles. In these cases, the anti-3MGA MAb might be useful to detect both 3MGA and compound **1** at the same time to find the body constitution causing pseudoaldosteronism when patients are administered licorice or GL.

## Methods

### Chemicals and reagents

GL was purchased from Calbiochem (San Diego, CA, USA). GA, astragaloside IV, and NAD^+^, EDC, 4-chloro-1-naphthol, and keyhole limpet hemocyanin (KLH) were obtained from Wako Pure Chemicals (Osaka, Japan). 3MGA, cortisone, cortisol, dexamethasone, *p*-hydroxybenzoic acid *n*-butyl ester (PHB), carboxymethylcellulose (CMC), penicillin and streptomycin, 2-morpholinoethanesulfonic acid (MES), and Clear-sol^®^ were purchased from Nacalai Tesque (Kyoto, Japan). Fetal bovine serum (FBS), subtilisin, Dulbecco’s modified Eagle’s medium, urethane, NHS, horseradish peroxidase (HRP)-labeled goat anti-mouse IgG antibody (Fc specific and whole molecule), human serum albumin (HSA), and bovine serum albumin (BSA) were obtained from Sigma-Aldrich (St. Louis, MO, USA). ABTS was obtained from Roche Applied Science, Penzberg, Upper Bavaria, Germany. [1,2,6,7-^3^H]cortisol (79.3 Ci/mmol), *p*-[glycyl-2-^3^H]-aminohippuric acid (4.56 Ci/mmol), and [6,7-^3^H(N)]estrone sulfate (51.8 Ci/mmol) were bought from Perkin Elmer (Waltham, MA, USA). Mustang^TM^ E positively charged PES membrane was obtained from Pall Co. (East Hills, NY, USA). Glass microfiber sheet (GF/A) was purchased from Whatman International (Maidstone, England). Skim milk was bought from Morinaga Milk Industry Co. Ltd. (Tokyo, Japan). Silica gel TLC plate (Silica gel F_254_) was purchased from Merck (Darmstadt, Germany). All other chemicals were of analytical grade or the highest grade available.

### Animals

Balb/c mice, Wistar rats, SD rats, and EHBRs were purchased from Japan SLC (Hamamatsu, Japan). EHBRs are known to express dysfunctional Mrp2 protein as a result of a point mutation in the open reading frame^20^. The animals received food and water ad libitum and were housed under controlled temperature (25 °C), humidity, and lighting (12 h light, 12 h dark) conditions. The experimental procedures were approved by the Animal Care Committee at the Graduate School of Pharmaceutical Sciences, Nagoya City University, Nagoya, in accordance with the guidelines of the Japanese Council on Animal Care.

### General Procedure

Optical rotation was recorded on a JASCO P-2100 polarimeter (Jasco Co., Tokyo, Japan). UV spectrum was recorded on a Shimadzu UV-1280 spectrophotometer (Shimadzu Co., Kyoto, Japan). ECD spectrum was recorded on a JASCO J-725 spectropolarimeter. IR spectrum was recorded on a Shimadzu IR affinity-1 spectrometer. NMR spectra were recorded on an Agilent Varian VNS500 spectrometer (Agilent Technologies, Santa Clara, CA, USA). Chemical shifts (ppm) were referenced to the residual solvent peaks (*δ*_H_ 3.31 and *δ*_C_ 49.0 for CD_3_OD). Negative-mode ESITOFMS was obtained on a JEOL JMS-T100LP AccuTOF LC-plus 4 G spectrometer using a sample dissolved in MeOH (Jeol Ltd., Tokyo, Japan). For LC-MS/MS analysis, Quattro Premier XE (Waters Co., Milford, MA, USA) system was used. Optical densities were measured using a microplate reader (ARVO MX 1420 multilabel counter, Perkin Elmer).

### Preparation of anti-3MGA MAb

To activate the hapten 3MGA for the preparation of anti-3MGA MAb, the 3MGA-KLH conjugate was synthesized by using a model based on the carbodiimide method with both EDC and NHS reagents^21^. 3MGA-HSA was also prepared using the same method, and was used for ELISA. We also evaluated the hapten number of the 3MGA-HSA conjugate by MALDI-TOF MS analysis. A broad peak coinciding with the 3MGA-HSA conjugate was detected around *m/z* 68,300 (data not shown). A molecular weight of approximately 66,400 for HSA revealed that the calculated value of the 3MGA component (MW = 646.8) was 2.94, indicating that approximately three molecules of 3MGA were coupled with one molecule of HSA. The MAb was developed by using the same methods described in our previous study^12^. Hybridomas secreting antibodies recognizing 3MGA were selected by ELISA and then subcloned 4 times by the limited dilution method. Anti-3MGA MAb was prepared successfully by using the method previously reported^12^. The heavy chain of the obtained MAb was classified using a Calbiochem mouse hybridoma subisotyping kit (EMD Biosciences, Darmstadt, Germany), whereas the light chain was estimated using an IsoQuick^TM^ mouse monoclonal isotyping strip (Sigma) according to the manufacturer’s instructions. The specificity of anti-3MGA MAb was examined by competitive ELISA according to Weiler and Zenk’s equation^22^, which was demonstrated as cross-reactivity (CR):

$${\rm{CR}}\,( \% )={{\rm{IC}}}_{{\rm{50}}}\,{\rm{for}}\,{\mathrm{3MGA}/\mathrm{IC}}_{{\rm{50}}}\,{\rm{for}}\,{\rm{compound}}\,{\rm{under}}\,{\rm{investigation}}\times {\rm{100}}$$

### ELISA system using anti-3MGA MAb

3MGA-HSA (1 µg/ml in 50 mM carbonate buffer, pH 9.6) was added into a 96-well plate (Nagel Nunc, Penfield, NY, USA) (100 µl/well), and incubated at 37 °C for 1 h. After the removal of 3MGA-HSA, the wells were washed with 200 µl/well PBS containing 0.05% Tween 20 (T-PBS) for 3 times. The wells were incubated with 300 µl/well of 5% skim milk (Morinaga-milk, Tokyo, Japan) at 4 °C overnight. After the wells were washed with T-PBS 3 times, the sample solution in H_2_O (50 µl/well) and anti-3MGA MAb (1:6,000 dilution with T-PBS) (50 µl/well) were added into the wells, and the plate was incubated at 37 °C for 1 h. After the wells were washed with T-PBS 3 times, HRP-labeled goat anti-mouse IgG (Fc specific) antibody (1:1,000 dilution with T-PBS) was added into the wells (100 µl/well), and the plate was incubated at 37 °C for 1 h. Next, the wells were washed with T-PBS 3 times, the substrate solution (0.006% H_2_O_2_ in 200 mM citrate buffer, pH 4.0/H_2_O/0.6 mg/ml ABTS 10:9:1) was added into the wells (100 µl/well), and incubated at room temperature for 15 min. The optical density at 405 nm was measured.

### Preparation of plasma and urine from EHBR orally treated with GA to check ELISA

Female EHBRs (8 or 9-week-old) were orally treated with GA (50 mg/kg) suspended in 0.5% CMC, and kept in metabolic cages. Blood samples (approximately 0.3 ml) were collected from the tail 1, 2, 3, 6, 12, 24, and 48 h after the oral treatment. Urine samples were collected 6, 12, 24, and 48 h after the oral treatment. The concentrations of 3MGA in the samples were measured by both ELISA and LC-MS/MS described in our previous paper^8^. Ten µl aliquots of plasma or urine from rats treated with GA, or standard solution of 3MGA in plasma or urine collected from normal rats were mixed with 20 µl of 1:1,000 diluted subtilisin, and incubated at 37 °C for 30 min. After adding 70 µl of EtOH containing 10 µg/ml of PHB used as internal standard, the solution was applied into the system. The mass spectrometer used an electrospray ionization source in the positive ion mode with multiple reaction monitoring (ESI(+)MS-MRM). The analytical column was an Inertsil ODS-4, 2 mm i.d. ×75 mm, 3 µm (GL Sciences Inc., Tokyo, Japan). The mobile phase was delivered using a linear gradient system, 0.5% AcOH (A): acetonitrile containing 0.5% AcOH (B), at a flow rate of 0.22 ml/min, with the following gradient profile: 30–55% B (0–1 min), 55–90% (1–3 min), 90% (3–6 min). The transitions (precursor to daughter) monitored and retention times were 647.4 to 453.5 m/z for 3MGA (4.4 min) and 195.2 to 139.0 *m/z* for PHB (4.8 min). Linear regression over the concentration range 3 nM–50 µM for 3MGA was examined using the peak-area ratio of the compounds to their internal standards and the least-squares method (*r*^2^ > 0.98).

### Eastern blot analysis

Five female EHBRs (7-week-old) were reared using GA suspension (1 mg/ml in 0.5% CMC) in drinking water in metabolic cages for 3 monthss, and the urine was pooled and kept at −20 °C until use.

The samples were spotted at base line on a TLC plate, and spreaded out using H_2_O/BuOH/AcOH (2:7:3). After drying, the developed TLC plate was sprayed with a blotting solution mixture of isopropanol-methanol-water (1:4:10) containing 0.1% NH_4_OH. The plate was placed onto a stainless-steel base and covered with a sheet of PES membrane followed by a glass microfiber filter sheet. The whole assembly was pressed evenly for 60 sec with a hot plate (120 °C). Afterwards, the PES membrane was separated from the TLC plate and air dried.

After drying, the blotted PES membrane was dipped into 0.1 M MES buffer (pH 4.7) containing 2% EDC and 1% NHS and kept at room temperature for 1 h to activate the carboxyl groups of 3MGA. After washing with distilled water, PBS containing 1% BSA was added and shaken at room temperature for 2 h for fixation. Next, the membrane was blocked with PBS containing 5% skim milk and 0.1% Tween 20 at 4 °C overnight to reduce non-specific adsorption. After washing twice with T-PBS for 5 min, the membrane was incubated with anti-3MGA MAb and kept at room temperature for 3 h with constant agitation. The membrane was then washed 2 times with T-PBS for 5 min and treated with 1:500 diluted HRP-labeled goat anti-mouse IgG (whole molecule) antibody in T-PBS for 1 h with gentle shaking. Finally, the membrane was washed twice with T-PBS and once with PBS, and exposed to 1 mg/ml of freshly prepared 4-chloro-1-naphthol-0.03% H_2_O_2_ in PBS for 20 min at room temperature. The reaction was stopped by washing with distilled water, and the immunostained PES membrane was allowed to dry and was photographed immediately.

### Isolation of compound 1 guided by eastern blot

One liter of pooled urine collected from female EHBRs treated with GA described above (dried weight approximately 40 g) was evaporated under reduced pressure after filtration. The concentrated urine (400 ml) was partitioned with EtOAc and *n*-BuOH. The *n*-BuOH-soluble materials (34 g) were subjected to an ODS silica gel column chromatography (CH_3_CN/H_2_O/AcOH 1:8:1 → 6:3:1 → 1:0:0). A fraction (2.2 g) eluted with CH_3_CN/H_2_O/AcOH (6:3:1) was separated with silica gel column (CHCl_3_/MeOH/AcOH 86:4:10 → 50:40:10 and then 0:100:0). The fraction eluted with CHCl_3_/MeOH/AcOH (60:30:10) was further purified by C_18_ HPLC (COSMOSIL 5C_18_-ARII (Nakalai), 5 µm, 10 mm i.d. × 250 mm, solvent MeCN/H_2_O/TFA, 28:72:0.1, flow rate 2.5 ml/min, detection 254 nm) to afford compound **1** (3.5 mg, *t*_R_ 21.3 min) with the guidance of positive immunostaining of eastern blot analysis using anti-3MGA MAb.

Compound **1**: colorless amorphous solid; [α]_D_^23^ +33 (*c* 1.0, MeOH); UV (MeOH) *λ*_max_ 248 (*ε* 3784) nm; ECD (MeOH) *λ* (*Δε*) 229 (+8.2) nm; IR (ATR) *ν*_max_ 3275, 2943, 2835, 1743, 1658, 1412, and 1207 cm^−1^; ^1^H-NMR (CD_3_OD, 500 MHz) and ^13^C-NMR (CD_3_OD, 125 MHz), see Table 1; ESIMS *m/z* 741 [M-H]^−^ and 565 [M-H-C_6_H_8_O_6_]^−^; HRESIMS *m/z* 741.3153 [M-H]^−^ (calcd for C_36_H_53_O_14_S, 741.3156) and m/z 565.2829 [M-H- C_6_H_8_O_6_]^−^ (calcd for C_30_H_45_O_8_S, 565.2835).

18*β*-glycyrrhetinic acid (GA): ECD (MeOH) *λ* (*Δε*) 229 (+9.4) nm; ^1^H-NMR (CD_3_OD, 500 MHz) *δ* 5.58 (1H, s, H-12), 3.17 (1H, dd 5.0, 4.5 Hz, H-3), 2.72 (1H, ddd 13.5, 3.5, 3.5 Hz, H-1a), 2.46 (1H, s, H-9), 2.20 (1H, dd 13.5, 3.5 Hz, H-18), 2.15 (1H, ddd 13.5, 4.5, 4.5 Hz, H-16a), 1.96 (1H, m, H-21a), 1.88 (1H, m, H-15a), 1.86 (1H, m, H-19a), 1.74 (1H, m, H-7a), 1.72 (1H, m, H-19b), 1.69 (1H, m, H-2a), 1.64 (1H, m, H-6a), 1.54 (1H, m, H-2b), 1.48 (1H, m, H-6b), 1.45 (1H, m, H-7b), 1.42 (3H, s, H-27), 1.41 (1H, m, H-21b), 1.41 (1H, m, H-22a), 1.41 (1H, m, H-22b), 1.25 (1H, d 12.5 Hz, H-15b), 1.17 (3H, s, H-29), 1.15 (3H, s, H-26), 1.14 (3H, s, H-25), 1.06 (1H, m, H-16b), 1.02 (1H, m, H-1b), 1.00 (3H, s, H-23), 0.84 (3H, s, H-24), 0.80 (3H, s, H-28), 0.77 (1H, d 12.5 Hz, H-5) and ^13^C-NMR (CD_3_OD, 125 MHz) *δ* 202.6 (C-11), 180.5 (C-30), 172.9 (C-13), 128.9 (H-12), 79.4 (C-3), 63.1 (C-9), 56.2 (C-5), 49.9 (C-18), 46.7 (C-8), 44.9 (C-14), 44.6 (C-20), 42.4 (C-19), 40.3 (C-1a), 40.2 (C-4), 39.0 (C-22), 38.3 (C-10), 33.8 (C-7), 33.0 (C-17), 32.0 (C-21), 28.8 (C-29), 28.7 (C-23), 28.7 (C-28), 27.8 (C-2), 27.6 (C-15), 27.4 (C-16), 23.8 (C-27), 19.3 (C-26), 18.6 (C-6), 16.9 (C-25), 16.3 (C-24).

### Protein Assay

Protein concentrations in various samples were determined using the BCA^TM^ Protein Assay kit (Thermo Scientific, Rockford, IL, USA) with BSA as the calibration standard.

### Determination of ***in vitro*** 11*β*-HSD2 activity using rat kidney microsomes

Assays were conducted as described by Diederich *et al*.^23^ with slight modifications as described in our previous study^8^. Briefly, kidney microsome fractions were obtained from male Wistar rats (7-week-old). The microsome fraction (1.0 mg protein) was then incubated at 37 °C for 30 min in a reaction mixture containing 50 nM [1,2,6,7-^3^H] cortisol, 1 mM NAD^+^, and samples in 250 of 0.1 M phosphate buffer (pH 6.0). The reaction was terminated by adding 250 µl EtOH containing 1.25 mg cortisol and 1.25 mg cortisone. A 10 µl aliquot of the solution was spotted onto a silica gel 60 F_254_ plate and was developed with chloroform:MeOH (9:1). Spots corresponding to cortisone (*Rf* value, 0.43) were detected under UV light, scraped into a scintillation vial containing Clear-sol^®^, and the radioactivity was measured using a liquid scintillation counter (Hitachi Aloka Medical, Tokyo, Japan). The IC_50_ was calculated from the least square regression line made from 3 points that crossed 50% of the control logarithmic concentration values.

### Pharmacokinetic experiments of compound 1 in SD rats or EHBRs orally treated with GA

Female SD rats or EHBRs (9-week-old) were anesthetized by an intraperitoneal (*i.p*.) injection of urethane (1 g/kg) and their jugular veins were exposed. GA suspended in 0.5% CMC was then administered orally (200 mg/kg) to the unconscious rats, and blood samples were collected from the jugular vein; urine samples were collected using a metabolic cage at an appropriate time over a 12-h period. Aliquots (10 µl) of plasma or urine were mixed with 20 µl of subtilisin (0.91 U/ml), incubated at 37 °C for 30 min, followed by the addition of 70 µl of astragalloside IV solution (1 µg/ml in ethanol containing 0.5% formic acid), and kept at −20 °C for 30 min. After centrifugation (2 × 10^4^ *g* for 7 min), the concentrations of compound **1** and GA in the supernatant of the samples prepared from plasma and urine were measured using LC-ESI(+)MS/MS at the following conditions: column, ACQUITY UPLC HSS C18 (1.8 μm, 2.1 × 100 mm, Waters); mobile phase, 0.5% formic acid (A): acetonitrile containing 0.5% formic acid (B), at a flow rate of 0.22 ml/min, with the following gradient profile: 15–96% B (0–3 min) and 96–96% (3–5.5 min); the transitions (precursor to daughter) monitored and retention times, 743.4 to 567.5 *m/z* for compound **1** (2.9 min), 785.4 to 143.0 *m/z* for astragaloside IV (2.9 min), and 471.3 to 91.0 *m/z* for GA (5.2 min). Linear regressions over the concentration range 16 nM–50 µM for compound **1** and GA were examined using the peak-area ratio of the compounds to their internal standards and the least-squares method (*r*^2^ > 0.98).

### Uptakes of compound 1 by rat kidney slices and cells expressing OAT1 and 3

Three female and three male EHBRs (9-week-old, respectively) were injected with urethane (1 g/kg, *i.p*.), and orally treated with an aqueous GA aqueous suspension (200 mg/kg in 1% CMC). Twelve h after the treatment, blood was collected from the abdominal vena cava, and the plasma samples of each sex rat were pooled. The concentrations of compound **1** and GA in pooled plasma samples were measured using LC-MS/MS as described below.

Female SD rats (9-week-old) were treated with urethane (1 g/kg, *i.p*.), and their kidneys were collected. After removal of the capsule, the kidneys were sliced at approximately 0.5-mm thickness in the frontal section of the organ, using a tissue slicer (Natsume Seisakusyo, Tokyo, Japan) and then cut at the renal hilus into two halves. The slices were pre-incubated at either 4 or 37 °C for 15 min in 24-well plates with 0.3 ml of the incubation medium (137 mM NaCl, 5.4 mM KCl, 0.95 mM CaCl_2_, 0.81 mM MgSO_4_, 0.44 mM KH_2_PO_4_, 0.25 mM Na_2_HPO_4_, 5.5 mM glucose, 10 mM MES, adjusted to pH 5.5). Five ml of pooled female EHBR plasma samples was lyophilized, and dissolved in 5 ml of the incubation medium. After pre-incubation, the medium was removed, and EHBR plasma samples in the incubation medium (at either 4 or 37 °C, 0.3 ml/well) were added into wells, and the kidney slices were further incubated for 2 h at either 4 or 37 °C. After washing with PBS three times, the kidney slices were homogenated in PBS using sonication, and the concentrations of protein were measured as mentioned above. Aliquots (100 µl) of kidney homogenate, pooled plasma samples, or standard solution were mixed with 0.1 ml of subtilisin (0.91 U/ml), incubated at 37 °C for 30 min, followed by the addition of 0.8 ml of astragalloside IV solution (1 µg/ml in ethanol), and kept at −20 °C for 30 min. After centrifugation (2 × 10^4^ *g* for 7 min), the supernatants were transferred into new tubes, and dried up at 50 °C followed by lyophilization. The residue was dissolved in 0.1 ml of 50% acetonitrile containing 1% formic acid, and after centrifugation (2 × 10^4^ × *g* for 7 min), the concentrations of compound **1** and GA in the supernatant of the samples prepared from kidney slices were measured using LC-MS/MS described below.

MDCKII cells were cultured in Dulbecco’s modified Eagle’s medium containing 10% FBS, 100 U/ml penicillin, and 100 µg/ml streptomycin at 37 °C in 5% CO_2_. Plasmids carrying the cDNAs encoding human organic anion transporter (OAT) 1 and 3 were prepared using the pCI-neo mammalian expression vector, as described previously^24^. Using the plasmids, MDCKII cells stably expressing OAT1 and 3 were prepared as described previously^25^, and tested for their functional expressions by evaluating the transports of [^3^H]*p*-aminohippurate and [^3^H]estrone sulfate, respectively, as probe substrates.

For the transport assay, the cells were seeded in 24-well plates (1 × 10^5^ cells/well), and incubated for 24 h. The cells were pre-incubated for 15 min at 37 °C with 0.25 ml of the incubation medium described above. Two ml of pooled female or male EHBR plasma samples was lyophilized, and dissolved in 2 ml of 10 mM MES (pH 5.5). The cells were then incubated further at 37 °C for 15 min with 0.1 ml of pooled female or male EHBR plasma samples. The surface of the cells was washed 3 times with 0.5 ml of ice-cold PBS, and then the contents were extracted with 0.2 ml of ethanol. The cell residues were dissolved by 0.1 ml of 1 M NaOH, and after neutralization by adding 0.1 ml of 1 M HCl, the concentrations of protein were measured as mentioned above. A 200 µl aliquot of the extract or standard solutions were mixed with 0.8 ml of astragaloside IV (1 µg/ml in ethanol), and kept at −20 °C for 30 min. After centrifugation (2 × 10^4^ × *g* for 7 min), the supernatants were transferred into new tubes, and dried up at 50 °C followed by lyophilization. The residue was dissolved in 0.1 ml of 50% acetonitrile containing 1% formic acid, and after centrifugation (2 × 10^4^ × *g* for 7 min), the concentrations of compound **1** and GA in the supernatant of the samples were measured using LC-MS/MS described below.

LC-ESI(+)MS/MS analysis was conducted at the following conditions: column, Scherzo SM-C18 (3 μm, 3 mm i.d. ×100 mm, Imtakt, Kyoto, Japan); mobile phase, (A) 5 mM AcNH_4_, (B) 125 mM AcNH_4_/acetonitrole 1:4, at a flow rate of 0.3 ml/min, with the following gradient profile: A:B = 50:50–0:100 (0–3 min) and 0:100 (3–10 min); compound **1** (5.8 min), astragaloside IV (3.0 min), GA (8.7 min); the transitions monitored were the same as described in the previous section. Linear regressions over the concentration range 0.08–2 µM for compound **1** and 3.2 nM–2 µM for GA were examined using the peak-area ratio of the compounds to their internal standards and the least-squares method (*r*^2^ > 0.98).

### Binding assay of compound 1 to serum albumin

In total 100 µl of pooled plasma samples from female EHBRs described above were ultra-centrifugated using Centricut W-20 (Molecular weight cut off 2 × 10^4^, Kurabo Industries Ltd., Osaka, Japan) according to the manufacturer’s instruction, and the concentration of compound **1** in the filtrate was measured by using LC-MS/MS described in uptake study section.

### Statistics

Statistical analysis of the data included repeated one-way analysis of variance (ANOVA) for comparison of multiple groups for multiple data, and Student’s *t*-test for comparison of two independent groups by PASW Statistics (version 18, SPSS; IBM, Armonk, NY, USA). A probability value of less than 0.05 was considered to indicate statistical significance.
